# αvβ5 Integrin/FAK/PGC-1α Pathway Confers Protective Effects on Retinal Pigment Epithelium

**DOI:** 10.1371/journal.pone.0134870

**Published:** 2015-08-05

**Authors:** Murilo F. Roggia, Takashi Ueta

**Affiliations:** Department of Ophthalmology, Graduate School of Medicine and Faculty of Medicine, The University of Tokyo, Tokyo, Japan; Eye Hospital, Charité, GERMANY

## Abstract

**Purpose:**

To elucidate the mechanism of the induction of peroxisome proliferator-activated receptor γ coactivator-1α (PGC-1α) by photoreceptor outer segments (POS) and its effects on retinal pigment epithelium (RPE).

**Methods:**

PGC-1α upregulation by POS was confirmed in ARPE-19 cells and in RPE *ex vivo*. To elucidate the mechanism, siRNAs against β5 integrin, CD36, Mer tyrosine kinase (MerTK), and Atg5, blocking antibodies against CD36 and MerTK, and a specific inhibitor for focal adhesion kinase (FAK) were used. We examined the effect of POS-induced PGC-1α upregulation on the levels of reactive oxygen species (ROS), mitochondrial biogenesis, senescence-associated β-galactosidase (SA-β-gal) after H_2_O_2_ treatment, and lysosomal activity. Lysosomal activity was evaluated through transcriptional factor EB and its target genes, and the activity of cathepsin D. Lipid metabolism after POS treatment was assessed using Oil Red O and BODIPY C11. RPE phenotypes of PGC-1α-deficient mice were examined.

**Results:**

POS-induced PGC-1α upregulation was suppressed by siRNA against β5 integrin and a FAK inhibitor. siRNAs and blocking antibodies against CD36 and MerTK enhanced the effect of POS on PGC-1α. The upregulation of PGC-1α increased the levels of mRNA for antioxidant enzymes and stimulated mitochondrial biogenesis, decreased ROS levels, and reduced SA-β-gal staining in H_2_O_2_-treated ARPE-19 cells. PGC-1α was critical for lysosomal activity and lipid metabolism after POS treatment. PGC-1α-deficient mice demonstrated an accumulation of type 2 lysosomes in RPE, thickening of Bruch’s membrane, and poor choriocapillaris vasculature.

**Conclusions:**

The binding, but not the internalization of POS confers protective effects on RPE cells through the αvβ5 integrin/FAK/PGC-1α pathway.

## Introduction

Age-related macular degeneration (AMD) is a leading cause of legal blindness worldwide. The pathogenesis is associated with age-related abnormalities in the retinal pigment epithelium (RPE) and other closely related tissues, including Bruch’s membrane and choriocapillaris [1,2]. Physiologically, RPE cells coordinate with photoreceptors in the homeostasis of phototransduction. The absorption of light, nutritional trafficking, and degradation of photoreceptor outer segments (POS) are some of the essential roles of RPE cells. POS comprise stacks of phospholipid bilayer membranes; POS tips are constantly shed by photoreceptors and are phagocytosed by RPE cells. In rhesus monkey, approximately 3000 disks are shed daily from 30 photoreceptors in each RPE cell, predominantly in the morning [3]. The shed POS need to be efficiently metabolized in RPE cells [4–7]. Furthermore, POS are bound and recognized at the apical surface of RPE cells; this process is mediated by αvβ5 integrin. Cluster of differentiation 36 (CD36) and Mer tyrosine kinase (MerTK) receptors are involved in POS internalization, leading to the digestion of the segments via the autophagy/lysosomal pathway [4,5,7–9].

In RPE, improperly degraded lipid byproducts accumulate as lipofuscin, which is a hallmark of senescent RPE cells [1,9]. Phagocytosed POS have been shown an important lipid source for the formation of lipofuscin [1,10].

While the pathogenic effects of impaired POS metabolism in RPE cells have been well known, the physiological roles of POS in the integrity of RPE cells have been poorly understood. It has been reported that POS treatment of cultured ARPE-19 cells confers protection against oxidative stress-induced apoptosis [11]. Paradoxically, lipofuscin accumulates in RPE of mice lacking β5 integrin [12]. These observations might suggest that RPE cells physiologically utilize POS in anti-senescent protection.

We have previously reported that POS phagocytosis upregulates the expression of peroxisome proliferator-activated receptor gamma coactivator 1-alpha (PGC-1α) in undifferentiated ARPE-19 cells [13]. However, the mechanism and roles of this upregulation remain unclear.

## Experimental Procedures

### Animals

All procedures were performed in accordance with the Association for Research in Vision and Ophthalmology Statement for the Use of Animals in Ophthalmic and Vision Research and were approved by the Institutional Animal Research Committee of the University of Tokyo. Mice were housed in a temperature-controlled room with access to fresh water and a rodent-specific diet. The animals were maintained under a 12-h light/dark cycle. PGC-1α-deficient mice were purchased from Jackson Laboratory. For the *ex vivo* culture experiment, RPE/choroid flat-mounts from C57BL/6 mice were used. For the isolation of RPE cells, RPE/choroid eyecups were incubated in dispase solution at 1000 PU/ml for 25 min at 37°C to separate RPE cells from the choroid.

### POS treatment of ARPE-19 cells

The *in vitro* model of POS phagocytosis by RPE cells was in accordance with previously published methods [4–6,13–15]. POS were isolated under dim red light from normal porcine eyes obtained from a local slaughterhouse, as described previously [13]. Freshly prepared POS were used in all experiments. Differentiated and undifferentiated ARPE-19 cells were treated with POS at the concentration of 10 POS/cell. The cells were then incubated in Dulbecco’s modified Eagle Medium/Nutrient Mixture F-12 (DMEM/F-12; Life Technologies, Palo Alto, CA, USA) supplemented with 10% fetal bovine serum (FBS) and antibiotics (50 μg/mL streptomycin and 50 U/mL penicillin) at 37°C in an atmosphere of 5% CO_2_. To induce differentiation, ARPE-19 cells were cultured in laminin-coated transwells for 3 weeks in the same medium supplemented with 1% FBS [16,17] and antibiotics. Differentiation was evaluated by the formation of tight junctions using ZO-1 immunostaining and polygonal morphology depicted on ZO-1. The effects of siRNAs, blocking antibodies, and other reagents on the binding and internalization of POS was assessed based on a protocol established by Finnemann et al [18]. Briefly, ARPE-19 cells seeded on 96-well plates were treated with FITC-labeled POS. The first plate was for assessing the extent of total phagocytosis (i.e., binding + internalization) while the second plate was for assessing the extent of internalization. FITC of the bound POS was quenched by 10 min treatment with 0.4% trypan blue in PBS. Cells on both plates were fixed with ice-cold methanol for 5 min, washed, and read at excitation and emission wavelengths of 485 nm and 535 nm, respectively. The fluorescence intensity from internalized POS was subtracted from that from total POS to calculate the extent of POS internalization.

### Antibodies and reagents

The following antibodies were used in this study: ZO-1 (1:100; sc-10804, Santa Cruz Biotechnology, Santa Cruz, CA, USA), prohibitin (1:100; sc-28259, Santa Cruz), transcriptional factor EB (TFEB) (1:100; sc-11004, Santa Cruz), CD36 (2 μg/ml; ab23680, Abcam, Cambridge, MA, US), MerTK (3.44 μg/ml; ab52968, Abcam), PGC-1α antibody (1μg/ml; ST1202, Calbiochem, La Jolla, CA, USA), and β-actin (1:2000; A5316, Sigma-Aldrich, St. Louis, MO, USA). ARPE-19 cells were pretreated with anti-CD36 and anti-MerTK antibodies for 1 h before a 3-h POS treatment. Latex beads (0.6 μm, 10 beads/cell, LB6) and Arg–Gly–Asp (RGD) peptide (0.5 mM; A8052) were from Sigma–Aldrich. Further, ARPE-19 cells were pretreated with RGD peptide for 30 min before a 3-h POS treatment. The cells were also pretreated with focal adhesion kinase (FAK) inhibitor 14 (500 μM) (sc-203950, Santa Cruz) for 30 min. Cathepsin D activity assay kit (ab65302) was purchased from Abcam.

### Quantification of mRNA and mitochondrial DNA using real-time PCR

RNA was extracted from homogenized samples of ARPE-19 cells or isolated mouse RPE cells using Trizol reagent (Invitrogen, Carlsbad, CA, USA). RNA was reverse-transcribed to cDNA using Superscript III for RT-PCR (Invitrogen). Quantitative real-time PCR was performed using the Thermal Cycler Dice Real Time System (Takara Bio, Inc., Shiga, Japan) with SYBR Green qPCR SuperMix-UDG (Invitrogen). Values for each gene were normalized to the level of glyceraldehyde 3-phosphate dehydrogenase (GAPDH). The primer sequences used in this study were confirmed using Primer-BLAST (http://www.ncbi.nlm.nih.gov/tools/primer-blast/), as listed in S1 Table.

Relative mitochondrial DNA content was determined by the ratio of mitochondrial DNA (cytochrome b) to nuclear DNA (GAPDH). DNA was isolated from ARPE-19 cells using the Wizard SV Genomic DNA Purification System (Promega, Madison, WI, USA). The following primer sequences were used for real-time PCR of DNA samples: cytochrome b (Fwd: 5′-TATTCCTTCATGTCGGACGA-3′ and Rev: 5′-AAATGCTGTGGCTATGACTG-3′) and GAPDH (Fwd: 5′-CAAGGTCATCCATGACAACTTTG-3′ and Rev: 5′-ACCACAGTCCATGCCATCACTGCCA-3′).

### Western blot

Total protein extracts from ARPE-19 samples were separated by sodium dodecyl sulfate polyacrylamide gel electrophoresis and transferred to nitrocellulose membranes, which were then blocked with 5% nonfat dry milk in phosphate-buffered saline with 0.1% Tween-20 (PBS-T buffer). Samples were incubated with primary antibodies overnight at 4°C in PBS-T buffer. After washing, the membranes were incubated with horseradish peroxidase-labeled anti-mouse/rabbit/goat secondary antibodies (Amersham Biosciences, Chalfont St. Giles, UK) for 1 h. After washing, the membranes were developed with ECL Plus Western Blotting Detection Reagents (GE Healthcare, Piscataway, NJ, USA). The levels of proteins of interest were calculated by normalization to the level of β-actin.

### Knockdown experiment

Small interfering RNAs (siRNAs) designed to specifically knockdown PGC-1α (sc-38884, Santa Cruz), β5 integrin (sc-35680, Santa Cruz), CD36 (sc-29995, Santa Cruz), MerTK (sc-37127, Santa Cruz), or Atg5 (sc-41445, Santa Cruz) were transfected into ARPE-19 cells using Lipofectamine RNAiMAX Transfection Reagent (Life Technologies), according to the manufacturer’s instructions. These siRNAs comprised three or more different sequences to minimize off-target effects. Transfection with negative control scramble siRNA (sc-37007, Santa Cruz) was used as the control. Experiments were conducted 48 h after siRNA transfection.

### Intracellular reactive oxygen species

The levels of reactive oxygen species (ROS) in ARPE-19 cells were determined after a 3-h POS treatment using the fluorescent dye 2,7-dichlorodihydrofluorescein diacetate (H_2_DCFDA) (C6827, Molecular Probes, Eugene, OR, USA), according to the manufacturer’s instructions. Fluorescence in the wells without cells was measured and subtracted from all readings.

### Mitochondrial activity assay

The oxidation of NADH to NAD+ by complex I was analyzed after a 3-h POS treatment using the Mitochondrial Complex I Activity Assay Kit (AAMT001, EMD Millipore, Billerica, MA, USA).

### Senescence-associated β-galactosidase (SA-β-gal) assay

ARPE-19 cells were incubated with or without POS for 3 h and then treated with 100 μM H_2_O_2_ for 2 h to induce senescence [19]. After 24 h, the cells were fixed and stained with beta-galactosidase using an SA-β-Gal Kit (K320-250, BioVision, Milpitas, CA, USA), according to the manufacturer’s instructions. The average proportions of stained cells in three microscopic views from each well were calculated; three wells were used for each treatment group.

### Lipid peroxidation level

ARPE-19 cells were incubated with or without POS for 6 h and in the medium only for 22 h. After washing, the cells were incubated with 10 μM boron dipyrromethene (BODIPY) C11 fluorescence dye (D3861, Molecular Probes) for 30 min at 37°C and then rinsed. The plate was read at excitation and emission wavelengths of 485 nm and 535 nm, respectively.

### Electron microscopy

Mouse eyes were histologically processed, as previously described [20]. Moreover, 70-nm-thick transverse sections were cut through the central retina and mounted onto grids. The sections were stained with lead citrate and uranyl acetate. Images were obtained by transmission electron microscopy (TEM) (80 kV, model JEM-1200EX; JEOL Ltd., Tokyo, Japan) using a charge-coupled device digital camera (model VELETA; JEOL Ltd.).

### Oil Red O staining and immunohistochemistry

For Oil Red O staining, adherent ARPE-19 cells were fixed with 10% formalin, treated with 60% isopropanol for 5 min, and dried. The cells were then incubated with Oil Red O solution (O1391, Sigma–Aldrich) for 10 min at room temperature, washed, and examined under a microscope. For visualization of choriocapillaris in mice, paraffin-fixed sections were blocked in 1 mg/mL bovine serum albumin and incubated with biotinylated *Ulex europaeus* agglutinin-I (UEA-I, 1:50; Vector Laboratories, Burlingame, CA, USA). After 1 h, the sections were washed and incubated with Avidin Texas Red (1:100; Vector Laboratories). After a 30-min incubation, the sections were washed and coverslipped.

### Statistics

All statistical analyses were performed using JMP11 software (SAS Institute Inc., Cary, NC, USA). Two-tailed student’s *t*-test was used for comparisons between unpaired groups. One-way analysis of variance (ANOVA) followed by *post-hoc* Tukey’s test was used for comparisons of three or more groups. A *P* value <0.05 was regarded as statistically significant.

## Results

### POS upregulate PGC-1α through αvβ5 integrin and FAK

**Fig 1:**
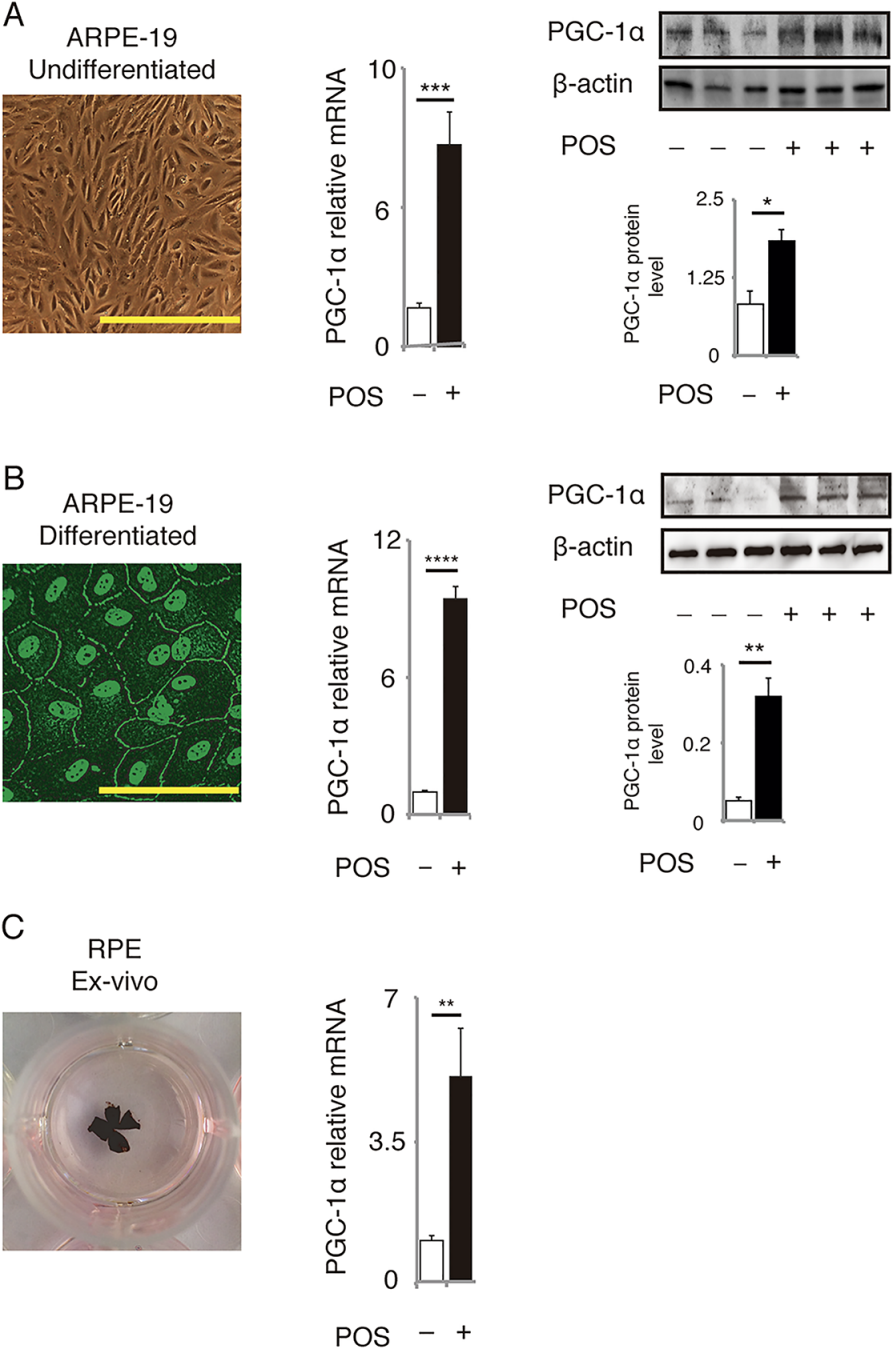
POS upregulate PGC-1α in RPE cells. PGC-1α mRNA and protein levels were upregulated in **(A)** undifferentiated ARPE-19 cells, **(B)** differentiated ARPE-19 cells, and **(C)** *ex vivo* RPE cells, treated with POS for 3 h for evaluation of mRNA and for 6 h for evaluation of protein levels. Morphology of differentiated ARPE-19 cells was confirmed by immunofluorescence for ZO-1 antibody. Mean ± SEM, n = 6–10 per group for mRNA level, n = 3 per group for protein level, two-tailed Student’s *t*-test, ***P < 0.001, ****P < 0.0001, **P < 0.01, *P < 0.05. Scale bars in the images of undifferentiated and differentiated ARPE-19 cells represent 100 μm and 200 μm, respectively.

We first tested whether the effect of POS treatment on PGC-1α expression in cultured RPE cells was equivalent under different conditions. The treatment increased PGC-1α mRNA and protein levels in undifferentiated ARPE-19 cells (Fig 1A). We observed the same effect in differentiated ARPE-19 cells (Fig 1B) as well as in *ex vivo* RPE culture (Fig 1C). Here, we used undifferentiated ARPE-19 cells to investigate PGC-1α-related functions in RPE cells.

**Fig 2:**
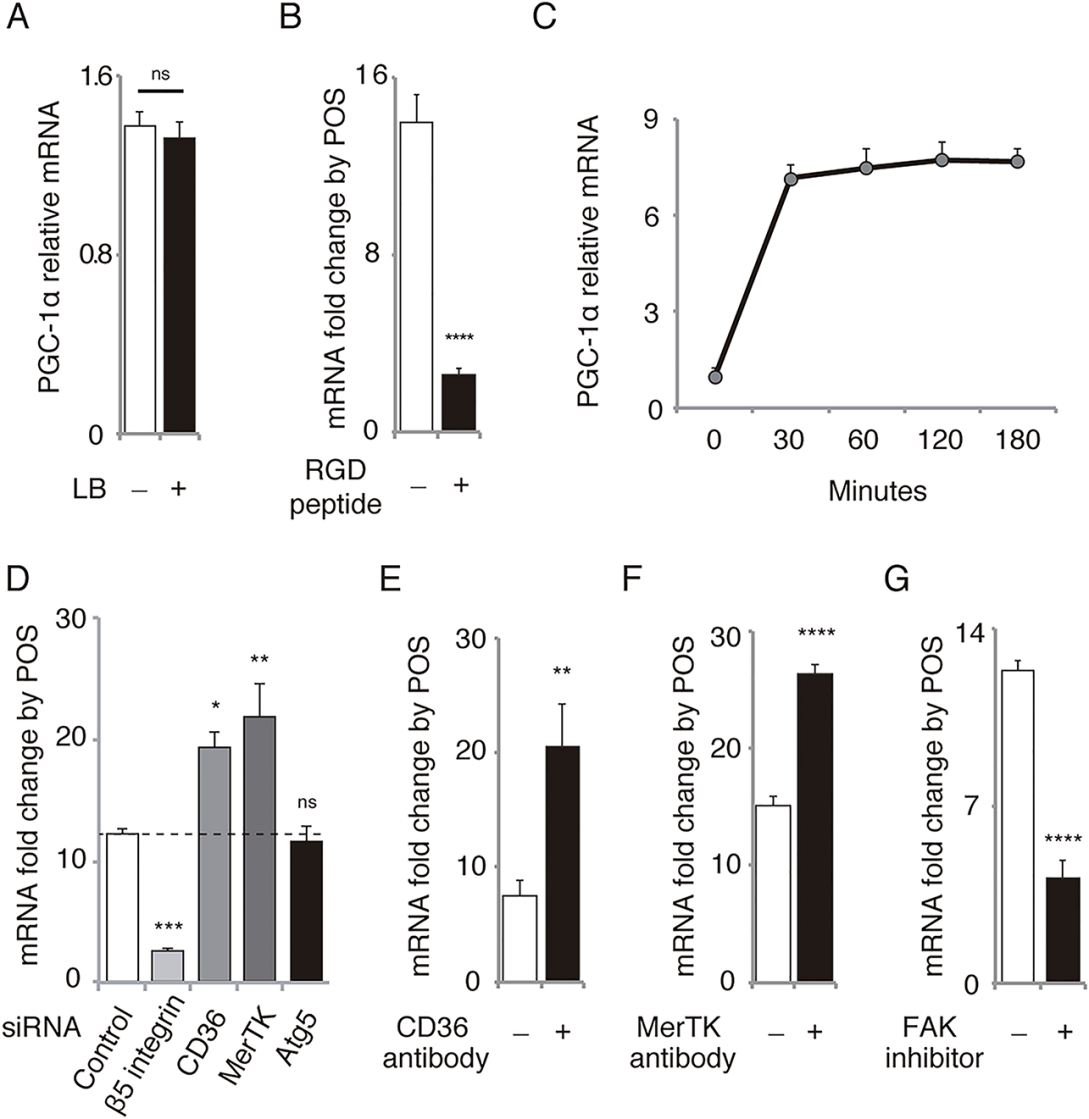
POS binding activates αvβ5 integrin/FAK/PGC-1α pathway in ARPE-19 cells. **(A)** PGC-1α mRNA level in cells was not influenced by the treatment with latex beads (LB). Mean ± SEM, n = 6 per group; two-tailed Student’s *t*-test; ns, not significant. **(B)** Upregulation of PGC-1α mRNA by POS treatment was significantly suppressed by pretreatment with RGD peptide. Mean ± SEM, n = 6 per group, two-tailed Student’s *t*-test, ****P < 0.0001. **(C)** Time course of PGC-1α mRNA level after POS treatment. **(D)** Upregulation of PGC-1α mRNA level by POS treatment was significantly suppressed by pretreatment with siRNA against β5 integrin, enhanced by siRNA against CD36 or MerTK, and not influenced by siRNA against Atg5. Mean ± SEM, n = 10 per group, two-tailed Student’s *t*-test, ****P < 0.0001, **P < 0.01, ***P < 0.001. **(E)** Upregulation of PGC-1α mRNA level by POS treatment was enhanced by pretreatment with CD36 antibody (2 μg/mL). Mean ± SEM, n = 6 per group, two-tailed Student’s *t*-test, *P < 0.05. **(F)** Upregulation of PGC-1α mRNA by POS treatment was enhanced by pretreatment with MerTK antibody (3.44 μg/mL). Mean ± SEM, n = 6 per group, two-tailed Student’s *t*-test, ****P < 0.0001. **(G)** Upregulation of PGC-1α mRNA by POS treatment was suppressed by pretreatment with FAK inhibitor 14 (500 μM). Mean ± SEM, n = 6 per group, two-tailed Student’s *t*-test, ****P < 0.0001.

To establish whether the effect was specifically associated with the phagocytosis of POS, we incubated the cells with latex beads used for the assessment of phagocytotic activity [21,22]. In contrast to POS treatment, treatment with latex beads did not increase PGC-1α mRNA (Fig 2A). To eliminate the possibility of other impurities affecting the results, we pretreated ARPE-19 cells for 30 min with RGD peptides, which inhibit POS binding [23] We observed that this pretreatment markedly suppressed the PGC-1α upregulation by POS (Fig 2B). Further, an increase in PGC-1α mRNA levels induced by POS is clearly visible as early as 30 min after POS treatment (Fig 2C). A report has revealed that POS internalization by RPE-J cells occurs after 90 min of the treatment [4]. Another recent study has reported that POS internalization by ARPE-19 and human fetal RPE cells occurs within 30 min after the treatment [15]. The stage of POS phagocytosis (i.e., binding, internalization, or digestion) specifically related to PGC-1α induction by POS remains unclear. To examine the possible scenarios, we first tested the effect of silencing of β5 integrin, CD36, MerTK, and Atg5 by RNA interference (Fig 2D) after confirming their efficiency (S1 Fig). In RPE cells where β5 integrin expression was silenced (i.e., binding was discouraged), PGC-1α upregulation by POS was markedly suppressed. However, when CD36 or MerTK was silenced (i.e., internalization was discouraged), PGC-1α upregulation was markedly enhanced rather than suppressed. PGC-1α upregulation by POS was not affected by Atg5 silencing (i.e., when digestion was discouraged). We also tested the consistency of the results using blocking antibodies against CD36 (2 μg/mL) and MerTK (3.44 μg/mL). In RPE cells pretreated with antibodies against CD36 (Fig 2E) or MerTK (Fig 2F) for 1 h, PGC-1α upregulation was not suppressed but enhanced. These results are consistent with those of a previous report showing that MerTK negatively controls POS binding by limiting the β5 integrin activity [14]. Our results implied that CD36 has similar effects on β5 integrin. If the binding/recognition of POS is associated with the PGC-1α upregulation, FAK, a major intracellular mediator of β5 integrin activation in RPE cells [5,12], may be involved in this signaling pathway. Therefore, we pretreated RPE cells with FAK inhibitor 14 for 30 min and incubated them with and without POS. The increase in PGC-1α mRNA levels caused by POS treatment markedly reduced in RPE cells pretreated with FAK inhibitor compared with controls (Fig 2G), suggesting that FAK is at least partly involved in the upregulation of PGC-1α by POS binding. Finally, we confirmed the effects of siRNAs, blocking antibodies, and other reagents on the binding and internalization of POS (S2 Fig): as expected, both binding and internalization was suppressed by β5 integrin siRNA and RGD peptide while only the internalization was suppressed by siRNAs and antibodies for CD36 and MerTK. Atg5 siRNA did not affect either binding or internalization of POS. FAK inhibitor 14 increased the POS binding significantly but with small extent while it drastically suppressed the internalization.

### POS elicit protective effects through PGC-1α in RPE cells

**Fig 3:**
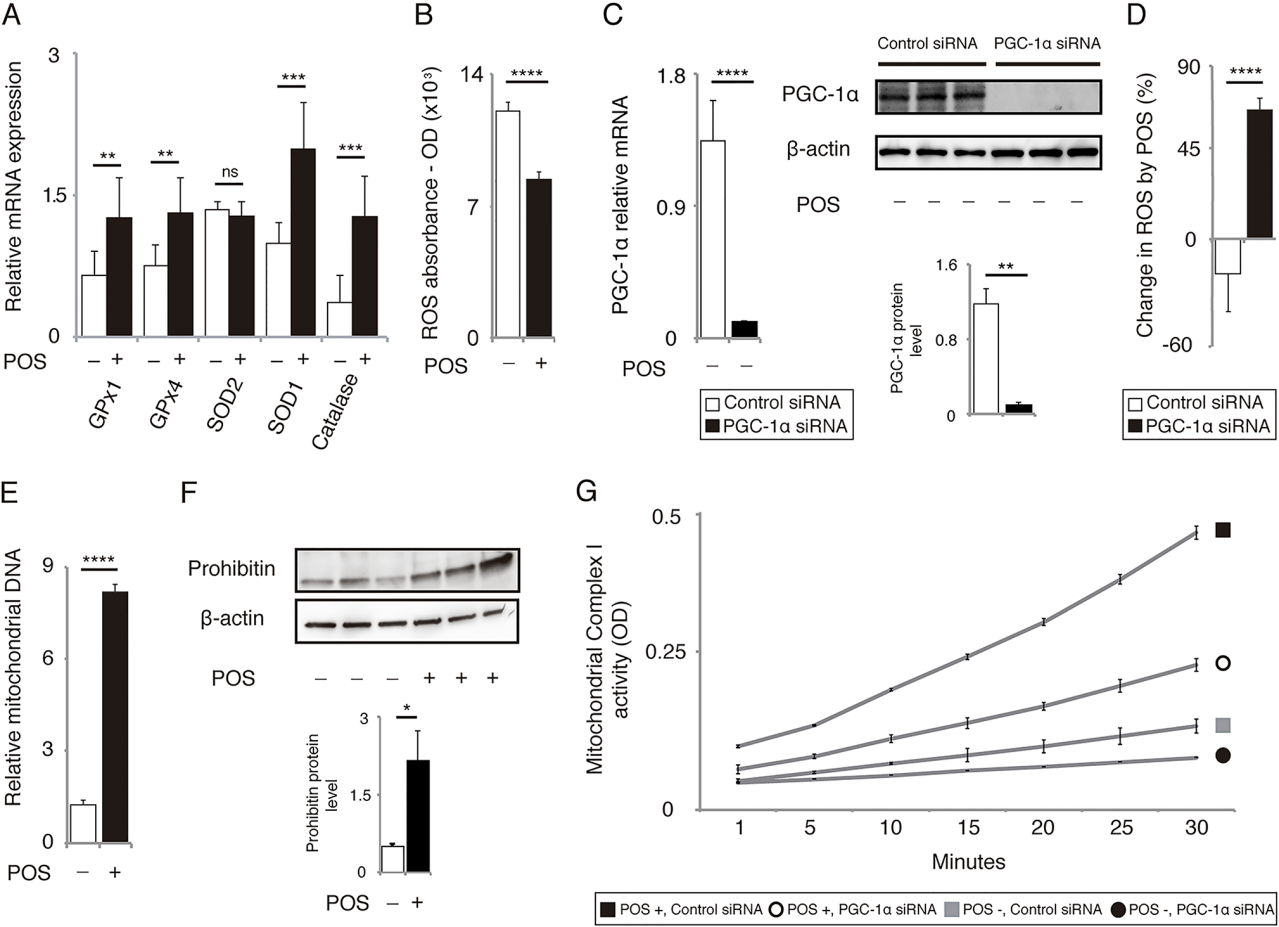
POS treatment decreases ROS level and upregulates mitochondrial biogenesis in ARPE-19 cells through PGC-1α. **(A)** POS treatment upregulated mRNA levels of anti-oxidant enzymes including GPx1, GPx4, SOD1 and catalase. Mean ± SEM, n = 6 per group, two-tailed Student’s *t*-test, ** *P* < 0.01, *** *P* < 0.001; ns, not significant. **(B)** POS treatment downregulated the Intracellular ROS levels evaluated by H_2_DCFDA. Mean ± SEM, n = 6 per group, two-tailed Student’s *t*-test, **P < 0.01, ***P < 0.001; ns, not significant. **(C)** Knockdown Efficacy of PGC-1α siRNA was confirmed by mRNA levels measured by RT-PCR (Mean ± SEM, n = 6 per group, two-tailed Student’s *t-*test, ****P < 0.0001) and protein levels by western blot analysis (mean ± SEM, n = 3 per group, two-tailed Student’s *t-*test, **P < 0.01). **(D)** POS treatment decreased ROS level in cells transfected with control siRNA, but rather increased in those transfected with PGC-1α siRNA. Mean ± SEM, n = 6 per group, two-tailed Student’s *t*-test, ****P < 0.0001. **(E)** POS treatment upregulated relative mitochondrial DNA content. Mean ± SEM, n = 6 per group, two-tailed Student’s *t*-test, ****P < 0.0001. **(F)** Western blot analysis showed the increased protein level of mitochondrial marker prohibitin in cells treated with POS. Mean ± SEM, n = 3 per group, two-tailed Student’s *t*-test, *P < 0.05. **(G)** POS treatment upregulated mitochondrial complex I activity in cells transfected with control siRNA, (n = 8 per group, Tukey’s test, *P* < 0.001), but not in those transfected with PGC-1α siRNA (n = 8 per group, Tukey’s test, P > 0.05).

PGC-1α has been known as a master regulator of mitochondrial biogenesis [24,25] and a potent suppressor of oxidative stress [26]. Increased PGC-1α expression in the muscle rescues age-related muscle wasting and metabolic decline in mice [27]. Therefore, we hypothesized that POS-induced PGC-1α upregulation increased mitochondrial biogenesis and decreased ROS production and conferred overall protective effects. We observed an increase in mRNA for the antioxidant enzymes glutathione peroxidase 1 (GPx1), GPx4, superoxide dismutase 1, and catalase (Fig 3A). The overall ROS level decreased in POS-treated RPE cells (Fig 3B). To investigate if the decrease in ROS depends on PGC-1α upregulation, we used PGC-1α siRNA after confirming its efficiency in ARPE-19 cells (Fig 3C). When PGC-1α was silenced by siRNA, POS treatment increased the ROS level in RPE cells (Fig 3D). This result indicates that PGC-1α may have an important role in POS-induced ROS reduction. POS treatment also increased the relative levels of mitochondrial DNA compared with nuclear DNA (Fig 3E). The level of prohibitin, a mitochondrial marker protein, also increased after POS treatment (Fig 3F), suggesting an increased mitochondrial volume. Functionally, POS treatment upregulated mitochondrial complex I activity, which was also abrogated by PGC-1α silencing (Fig 3G).

**Fig 4:**
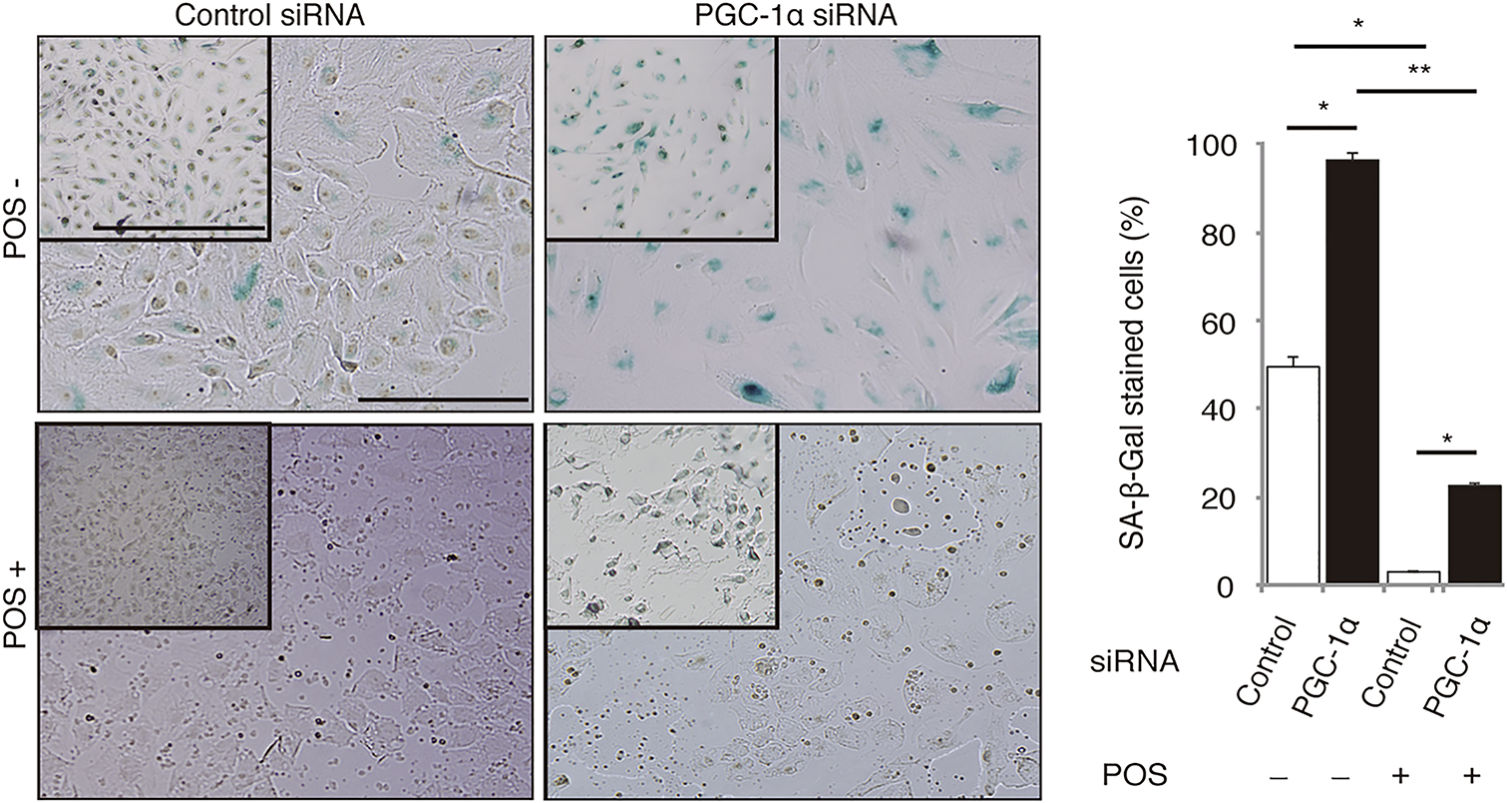
POS treatment rescues H_2_O_2_-induced SA-β-gal activity through PGC-1α. ARPE-19 cells were incubated with or without POS for 3 h and then treated with 100 μM H_2_O_2_ for 2 h to induce senescence. In cells transfected with control siRNA, POS treatment almost completely rescued SA-β-gal activity induced by H_2_O_2_. In contrast, in cells transfected with PGC-1α siRNA, H_2_O_2_ induced significantly stronger and more frequent SA-β-gal staining, and POS treatment did not rescue as much as it did in cells transfected with control siRNA. Mean ± SEM, n = 3 per group, ANOVA and Tukey’s test, *P < 0.05, **P < 0.01. Scale bars in large pictures and in insets are 100 μm and 500 μm, respectively.

We also evaluated the effects of POS and PGC-1α on SA-β-gal activity (Fig 4). We induced ARPE-19 cell senescence by incubation with 100 μM H_2_O_2_ for 2 h and conducted SA-β-gal staining 24 h later [19]. The 3-h POS pretreatment almost completely rescued H_2_O_2_-induced senescence in RPE cells transfected with control siRNA. In contrast, PGC-1α silencing aggravated H_2_O_2_-induced senescence and markedly suppressed the counteracting effect of POS (Fig 4).

### PGC-1α regulates lysosomal activity in RPE cells

**Fig 5:**
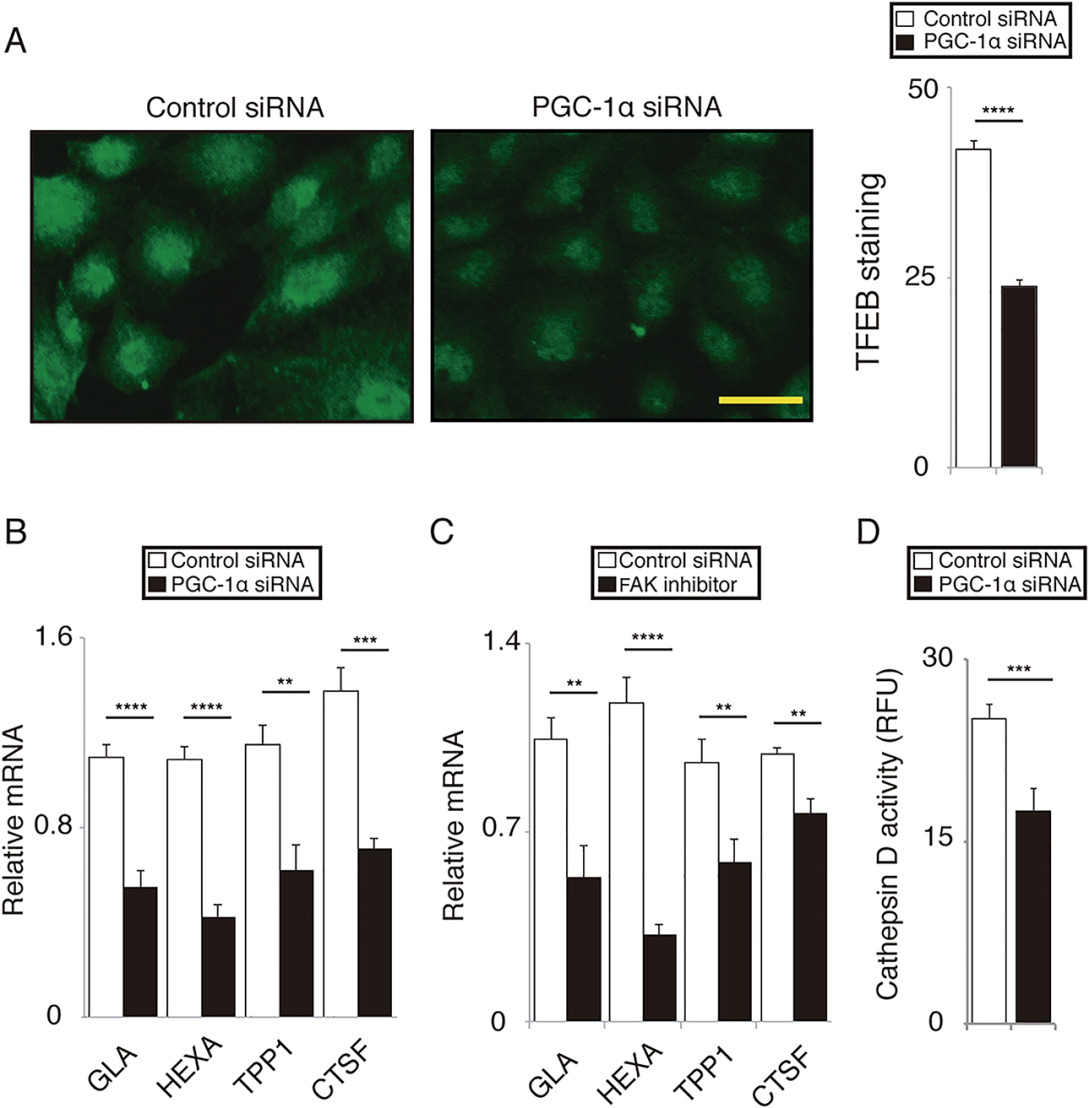
PGC-1α regulates lysosomal activity in ARPE-19 cells. **(A)** Nuclear immunofluorescence with TFEB antibody was weaker in cells transfected with PGC-1α siRNA compared to those transfected with control siRNA. Mean ± SEM, n = 6 per group, two-tailed Student’s *t*-test, ****P < 0.0001; scale bar, 50 μm. **(B)** In cells transfected with PGC-1α siRNA, mRNA levels of TFEB target genes including galactosidase α (GLA), hexosaminidase A (HEXA), tripeptidyl peptidase I (TPPI), and cathepsin F (CTSF) were downregulated compared to those transfected with control siRNA. Mean ± SEM, n = 6 per group, two-tailed Student’s *t*-test, **P < 0.01, ***P < 0.001, ****P < 0.0001. **(C)** In cells pretreated with FAK inhibitor 14, mRNA levels of TFEB target genes including GLA, HEXA, TPPI, and CTSF were downregulated compared to those pretreated with vehicle control. Mean ± SEM, n = 6 per group, two-tailed Student’s *t*-test, **P < 0.01, ****P < 0.0001. **(D)** In cells transfected with PGC-1α siRNA, cathepsin D activity was significantly downregulated compared to those transfected with control siRNA. Mean ± SEM, n = 6 per group, two-tailed Student’s *t*-test, ***P < 0.001.

The accumulation of peroxidized lipids and lipofuscin is a hallmark of aged RPE cells [1,6,7,9] and is associated with a decline in lysosomal activity [7,28]. Transcription factor EB (TFEB) is a master regulator of lysosomal activity [29,30]. TFEB and PGC-1α cooperatively regulate lysosomal activity and lipid catabolism in Huntington’s disease mice model [31] and under starvation [32]. We speculated that the αvβ5 integrin/FAK/PGC-1α pathway may be involved in lysosomal activity, facilitating POS metabolism in RPE cells. Furthermore, we observed that when PGC-1α was silenced, nuclear staining of RPE cells with TFEB antibody was markedly weaker than in the cells transfected with control siRNA (Fig 5A). PGC-1α silencing reduced mRNA levels of TFEB target genes encoding galactosidase α, hexosaminidase A, tripeptidyl peptidase I, and cathepsin F (Fig 5B). mRNA levels of these genes were also downregulated by FAK inhibitor 14 (Fig 5C). Moreover, PGC-1α silencing markedly decreased cathepsin D activity (Fig 5D), a major cathepsin involved in the degradation of rhodopsin and POS [6,9,33].

**Fig 6:**
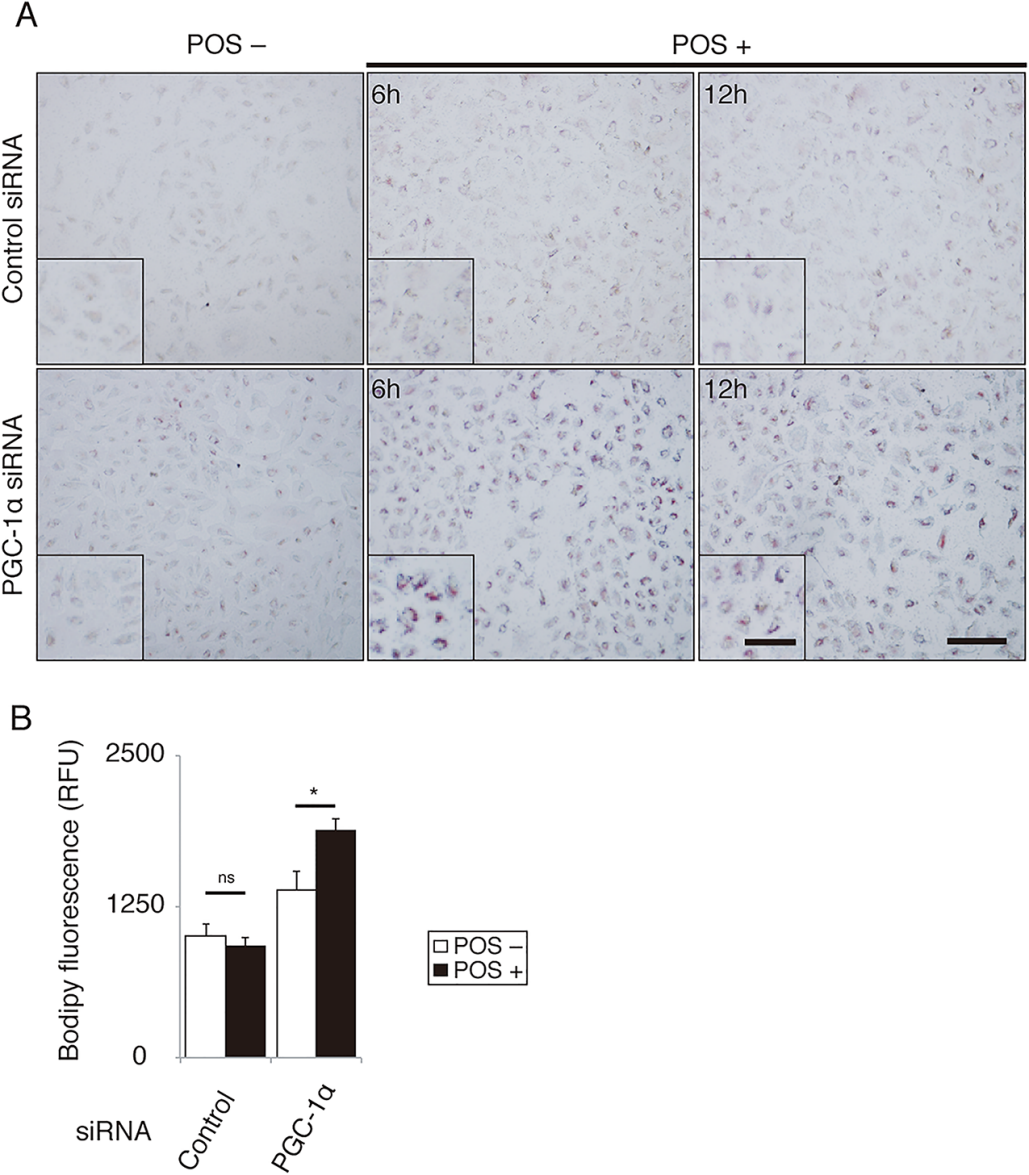
PGC-1α facilitates lysosomal degradation of POS in ARPE-19 cells. **(A)** In cells transfected with PGC-1α siRNA, Oil Red O staining after POS treatment was more intense than those transfected with control siRNA. Scale bar, 200 μm. **(B)** In cells transfected with PGC-1α siRNA, the accumulation of peroxidized lipids evaluated by BODIPY C11 fluorescence increased after POS treatment compared to those transfected with control siRNA. Mean ± SEM, n = 6 per group, ANOVA and Tukey’s test, *P < 0.05.

These data suggest the importance of PGC-1α for lysosomal activity in RPE cells. If the αvβ5 integrin/FAK/PGC-1α pathway were not sufficiently activated, RPE cells would not efficiently catabolize POS, leading to an abnormal lipid accumulation. Following this, we evaluated the role of PGC-1α in POS degradation. Using Oil Red O staining, we compared intracellular lipid accumulation in cells transfected with PGC-1α siRNA and control siRNA before and after POS treatment. The staining was more intense in ARPE-19 cells whose PGC-1α was silenced than in controls before and after 6 and 12 h of POS treatment (Fig 6A). We also examined the accumulation of peroxidized lipids after POS treatment using BODIPY C11 dye (Fig 6B). The results revealed that PGC-1α silencing approximately doubled the accumulation of peroxidized lipid in POS-treated RPE cells.

### Senescence-related phenotypes in RPE of PGC-1α-deficient mice

**Fig 7:**
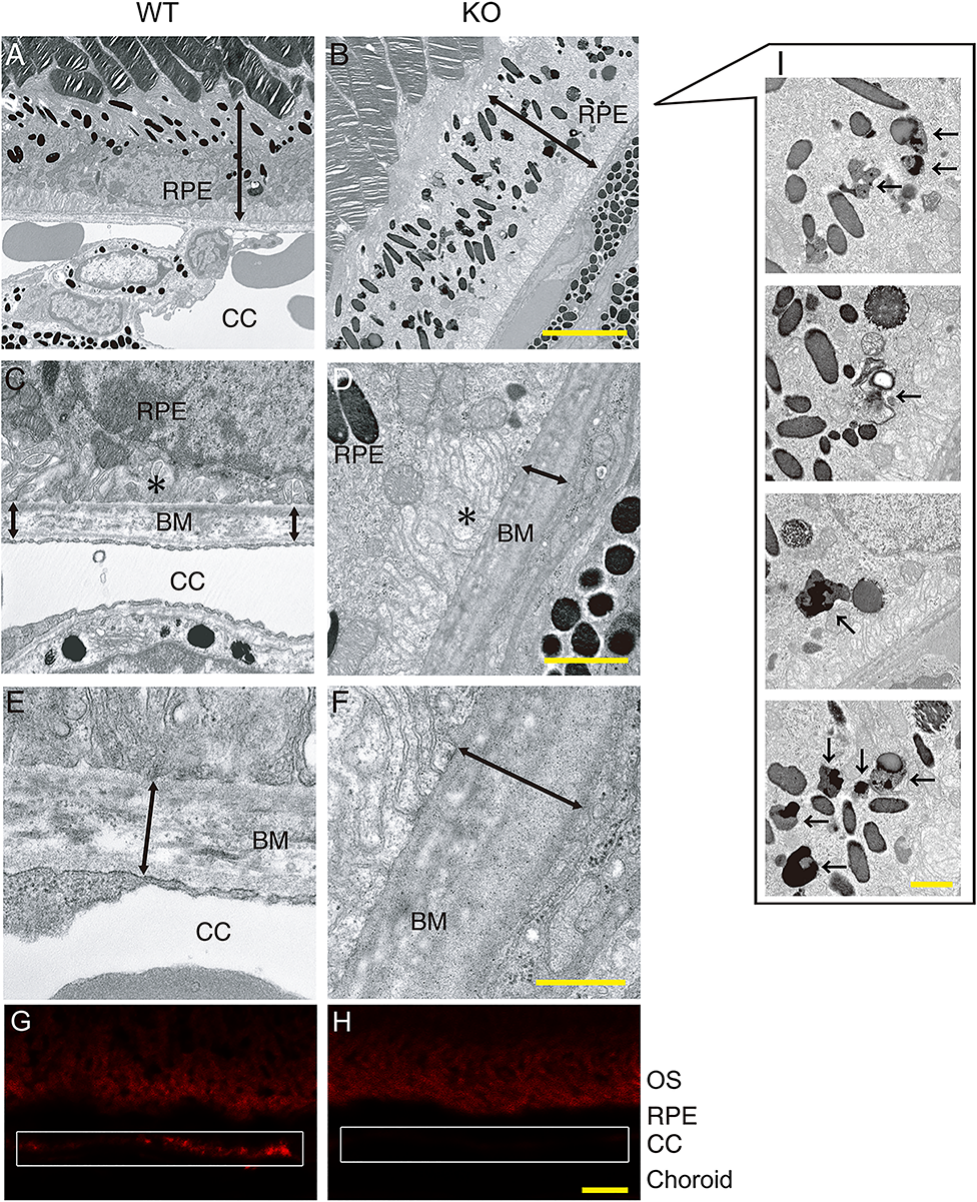
Accelerated senescence is observed in RPE of PGC-1α-deficient mice on TEM. **(A, B)** The number of melanolysosomes is increased in the RPE of PGC-1α-deficient mice, especially at the basal area. CC; choriocapillaris; scale bar, 5 μm. **(C, D)** Bruch’s membrane (BM), CC, and basal infoldings of RPE (asterisks). Note that BM of the PGC-1α-deficient mice is thicker with increased electron density than that of the control mice. CC is not well observed in PGC-1α-deficient mice, while it is abundant in the control mice. Loss of truncation of basal infoldings is evident in PGC-1α-deficient mice. Scale bar, 1 μm. **(E, F)** Magnified view of BM and CC. Scale bar, 500 nm. **(G, H)** CC is poorly depicted by UEA-I staining in PGC-1α-deficient mice compared to the control mice. CC is well depicted in a dot-like pattern corresponding to the capillaries in WT mice. Scale bar, 5 μm. **(I)** Magnified views of various type 2 lysosomes in RPE cells of PGC-1α-deficient mice (black arrows). Scale bar, 1 μm.

To further elucidate the role of PGC-1α in lipid metabolism and senescence in RPE cells, phenotypes of 6-month-old PGC-1α-deficient mice and age-matched control littermates were compared. We examined the phenotypes of RPE, Bruch’s membrane, and choriocapillaris (the original site of AMD). The retina of PGC-1α-deficient mice demonstrates regular morphology and function [34]. In contrast, our TEM examination revealed an abundant accumulation of melanolysosomes, especially type 2 lysosomes (Fig 7B and 7I vs. 7A) and loss of truncation of basal infoldings (Fig 7D vs. 7C) in RPE cells. Type 2 lysosomes [35] and loss of truncation of basal infoldings [9] are considered associated with aging due to impaired lysosomal activity. We also observed the thickening of the Bruch’s membrane (Fig 7D vs. 7C; Fig 7F vs. 7E) and scarcity of choriocapillaris (Fig 7B vs. 7A) in 6-month-old PGC-1α-deficient mice compared with controls. These phenotypes are also associated with aging RPE cells [1,36–38]. Staining with UEA-I lectin confirmed poor choriocapillaris vasculature in PGC-1α-deficient mice compared with controls (Fig 7H vs. 7G).

## Discussion

**Fig 8:**
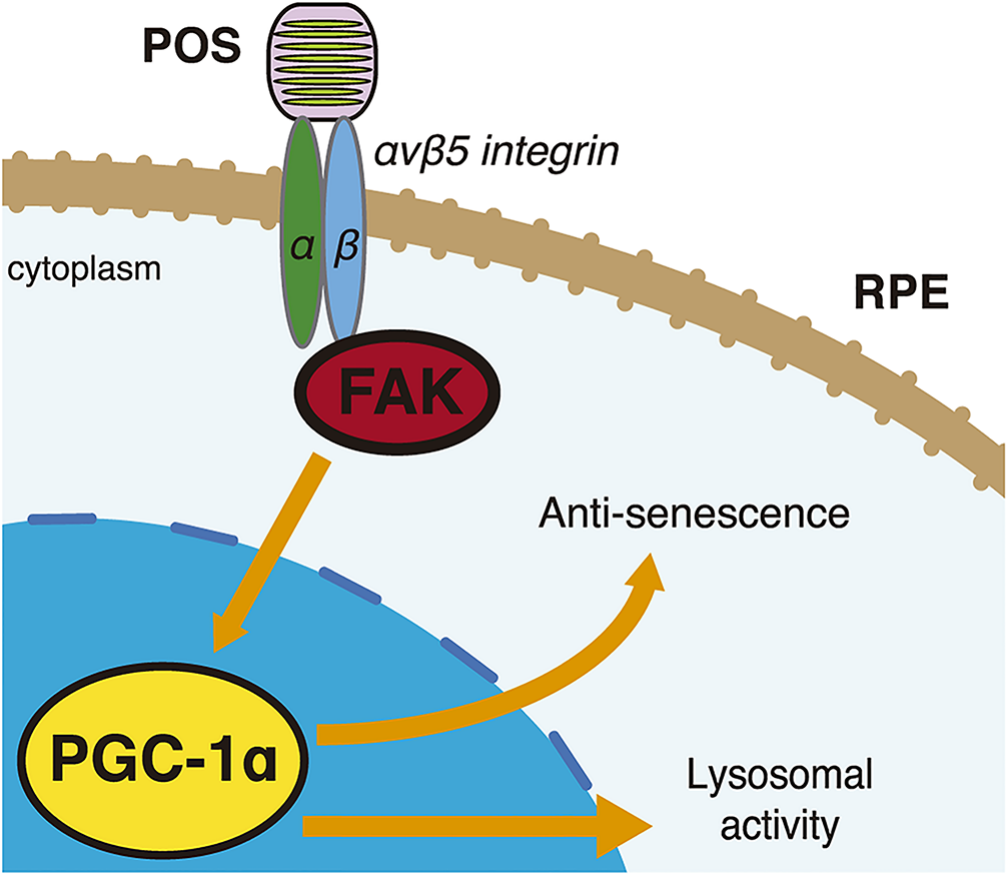
Schematic drawing of the pathway shown in the present study. In RPE cells POS binding activates αvβ5 integrin/FAK/PGC-1α pathway, which confers protections and facilitates lysosomal activity.

Here, we examined the role of PGC-1α in POS degradation. We demonstrated that an inherent αvβ5 integrin/FAK/PGC-1α pathway in RPE cells is activated by the binding, but not the internalization, of POS. This process confers anti-oxidative protection and facilitates lysosomal degradation (Fig 8). Our results explain the previously reported observation that RPE cells become more susceptible to oxidative stress [11] and aging [12] when they do not actively phagocytize POS.

PGC-1α has been known as a powerful regulator of transcription factors for mitochondrial biogenesis (such as mitochondrial transcription factor A [TFAM]), fatty acid oxidation (such as peroxisome proliferator-activated receptors [PPARs]), suppression of oxidative stress (such as nuclear transcription factor 2 [NRF2]) and angiogenesis (such as estrogen-related receptors, [ERRs]) [39–41], although roles of PGC-1α in RPE have not been fully understood. RPE cells combat the high oxygen consumption, light absorption, and lipid flux through POS phagocytosis, all of which can result in considerable oxidative and metabolic stress [7]. Numerous studies have suggested that oxidative stress and aging affect POS phagocytosis. For instance, sublethal exposure to H_2_O_2_ can inhibit FAK activation in ARPE-19 cells [42] and photo-oxidative stress suppresses β5 integrin expression [43]. ARPE-19 cells cultured on Bruch’s membrane from aged donors have lower POS-phagocytic activity than those cultured on the membrane from young donors [44]. Our results suggest that the αvβ5 integrin/FAK/PGC-1α pathway has an important protective function in RPE cells and that the activity of the pathway declines with age.

We identified accelerated aging phenotypes in RPE, Bruch’s membrane, and choriocapillaris of PGC-1α-deficient mice. The loss of choriocapillaris with age and in the early stage of AMD has been confirmed in humans [35] Moreover, loss of choriocapillaris and thickening of the Bruch’s membrane hampers nutrient supply to RPE cells and metabolite release from those cells, leading to RPE loss (i.e., dry AMD). In contrast, the loss of choriocapillaris may be counteracted by neoangiogenesis, leading to choroidal neovascularization (i.e., wet AMD) [35]. Dry and wet AMD are the late stages of AMD, wherein the degeneration cannot be fully reversed. Choriocapillaris is maintained by VEGF secretion from RPE cells; the results of our previous study reveal that PGC-1α is an important regulator of VEGF expression in RPE cells, particularly in response to POS phagocytosis [13]. Therefore, a therapeutic approach targeting PGC-1α at the early stage of AMD may suppress the progression of this disease. For example, several compounds have been reported as inducers of PGC-1α. These include pyrroloquinoline quinone [45], AMP-activated protein kinase activators such as 5-aminoimidazole-4-carboxamide ribonucleotide [46], and adiponectin receptor agonists [47].

One limiting aspect of our study was the use of ARPE-19 cells for investigating the mechanism and effects of POS-induced PGC-1α overexpression in RPE cells. However, the experimental conditions were in accordance with methodologies that have established the physiology and pathology of RPE cells [4–6,13–15]. ARPE-19 cells in various culture conditions have been demonstrated to use the MFG-E8/αvβ5 integrin/FAK mechanism to recognize and bind POS [23,42,43,48,49] In addition, we tested the consistency of the PGC-1α upregulation in differentiated ARPE-19 cells and *ex vivo* RPE culture and examined the phenotypes of PGC-1α-deficient mice.

In conclusion, we have found that the αvβ5 integrin/FAK/PGC-1α pathway is involved in POS-induced PGC-1α upregulation, which confers protective effects on RPE cells.

## Supporting Information

S1 Fig Knockdown efficacy of siRNAs for β5 integrin, CD36, MerTK, and Atg5 in ARPE-19 cells. **(A)** β5 integrin mRNA levels measured by RT-PCR. Mean ± SEM, n = 6 per group, two-tailed Student’s *t-*test, ****P < 0.0001. β5 integrin protein levels measured by western blot analysis. Mean ± SEM, n = 3 per group, two-tailed Student’s *t-*test, ***P < 0.001. **(B)** CD36 mRNA levels measured by RT-PCR. Mean ± SEM, n = 6 per group, two-tailed Student’s *t-*test, ***P < 0.001. CD36 protein levels measured by western blot analysis. Mean ± SEM, n = 3 per group, two-tailed Student’s *t-*test, ***P < 0.001. **(C)** MerTK mRNA levels measured by RT-PCR. Mean ± SEM, n = 6 per group, two-tailed Student’s *t-*test, ****P < 0.0001. MerTK protein levels measured by western blot analysis. Mean ± SEM, n = 3 per group, two-tailed Student’s *t-*test, ***P < 0.001. **(D)** Atg5 mRNA levels measured by RT-PCR. Mean ± SEM, n = 6 per group, two-tailed Student’s *t-*test, ***P < 0.001. Atg5 protein levels measured by western blot analysis. Mean ± SEM, n = 3 per group, two-tailed Student’s *t-*test, ***P < 0.001.
(EPS)

S2 Fig The effects of siRNAs, blocking antibodies, and other reagents on the binding and internalization of POS in ARPE-19 cells. **(A)** Fluorescence intensities showing the extent of POS binding on ARPE-19 cells transfected with siRNAs. Mean ± SEM, n = 8 per group, ANOVA and Tukey’s test, **P < 0.01. **(B)** Fluorescence intensities showing the extent of POS internalization in ARPE-19 cells transfected with siRNAs. Mean ± SEM, n = 8 per group, ANOVA and Tukey’s test, ***P < 0.001. **(C)** Fluorescence intensities showing the extent of POS binding on ARPE-19 cells treated with various reagents and vehicle control. Mean ± SEM, n = 8 per group, ANOVA and Tukey’s test, ***P < 0.001. **(D)** Fluorescence intensities showing the extent of POS internalization in ARPE-19 cells treated with various reagents and vehicle control. Mean ± SEM, n = 8 per group, ANOVA and Tukey’s test, ***P < 0.001.
(EPS)

S1 Table Primer sequences used for real-time RT-PCR. (DOCX)
