# Supplementary material for: αvβ5 Integrin/FAK/PGC-1α Pathway Confers Protective Effects on Retinal Pigment Epithelium
Source: PLoS One. 2015 Aug 5;10(8):e0134870. doi: 10.1371/journal.pone.0134870 (PMC4526642; doi:10.1371/journal.pone.0134870)
Supplement: S1 Table — (DOCX) [file pone.0134870.s003.docx]

| Genes | Forward | Reverse |
| --- | --- | --- |
| Mouse GAPDH | CACATTGGGGTAGGAACAC | AACTTTGGCATTGTGGAAGG |
| Mouse PGC-1α | AATGCAGCGGTCTTAGCACT | GTGTGAGGAGGGTCATCGTT |
| Human GAPDH | TTGATTTTGGAGGGATCTCG | GAGTCAACGGATTTGGTCGT |
| Human PGC-1α | GTGAAGACCAGCCTCTTTGC | TCACTGCACCACTTGAGTCC |
| Human GPx1 | CTCTTCGAGAAGTGCGAGGT | TCGATGTCAATGGTCTGGAA |
| Human GPx4 | GCACATGGTTAACCTGGACA | CTGCTTCCCGAACTGGTTAC |
| Human SOD1 | TGGCCGATGTGTCTATTGAA | GGGCCTCAGACTACATCCAA |
| Human SOD2 | TCCACTGCAAGGAACAACAG | TCTTGCTGGGATCATTAGGG |
| Human catalase | GCCTGGGACCCAATTATCTT | GAATCTCCGCACTTCTCCAG |
| Human HEXA | GTCATTGAATACGCACGGCT | GACTGGGATTCACTGGTCCA |
| Human TPPI | GATGTGGCTGCACTTTCTGA | AGCCACGGGTTACATCAAAG |
| Human GLA | CATCAGCCCTCAAGCCAAAG | ACCAATCTCCTGCCGGTTTA |
| Human CTSF | GCCTGTCCGTCTTTGTCAAT | TTGTTGCCAGGCTCTTTCCT |
